# Two types of microorganisms isolated from petroleum hydrocarbon pollutants: Degradation characteristics and metabolic pathways analysis of petroleum hydrocarbons

**DOI:** 10.1371/journal.pone.0312416

**Published:** 2024-11-13

**Authors:** Xiafei Yin, Xin Wang, Minjun Qiu, Wei Shao, Min Ai, Guobin Liang

**Affiliations:** 1 School of Resources and Environmental Engineering, Jiangsu University of Technology, Changzhou, P. R. China; 2 Consulting Department, Jiangsu Longhuan Environmental Technology Co., LTD, Changzhou, P. R. China; 3 Consulting Department, Jiangsu Longheng Environmental Technology Co., LTD, Changzhou, P. R. China; 4 School of Chemistry and Chemical Engineering, Jiangsu University of Technology, Changzhou, P. R. China; Universidad Tecnica de Manabi, ECUADOR

## Abstract

The petroleum hydrocarbons in seawater have been worldwide concern contaminants. Biological method, with the advantages of low cost, minimal environmental impact, and no secondary pollution, is a promising method for petroleum hydrocarbon treatment. In this study, two strains, identified as *Stenotrophomonas acidaminiphila* and *Ochrobactrum*, were demonstrated to possess the ability to degrade petroleum hydrocarbons. The mixed culture composed of *Stenotrophomonas acidaminiphila* and *Ochrobactrum* at a 2:1 ratio was able to achieve 79.41% degradation of the total petroleum hydrocarbons after 5 days. Besides, the average removal efficiencies of C10-C30 components in petroleum hydrocarbons by *Stenotrophomonas acidaminiphila*, *Ochrobactrum*, and mixed culture were 62.98%, 59.14% and 73.30%, respectively. The possible degradation pathways of petroleum hydrocarbons had been speculated through gas chromatography-mass spectrometry (GC-MS) and differential gene expression metabolomics analyses. The toxicity of products from the biodegradation of petroleum hydrocarbons was greatly reduced.

## Introduction

With the rapid development of the economy and society, the one-time energy consumption of China is substantial, with oil accounting for the largest proportion. Simultaneously, oil production, storage, and transportation are becoming increasingly developed, leading to frequent offshore oil spills and serious pollution of the marine environment. According to the 2018 China Marine Ecological Environment Status Bulletin released by the Ministry of Ecology and Environmental Protection, the area of the four types of poor water quality sea areas in the summer of 2018 was approximately 33000 km^2^, with the main exceeding substances being active phosphates, inorganic nitrogen, and petroleum. Petroleum hydrocarbons are a mixture of various hydrocarbons, such as n-alkanes, branched alkanes, cycloalkanes, and aromatics, and a small amount of other organic substances, such as sulphides, nitrides, and cycloalkanes, 95% of which comprise carbon and hydrogen compounds [1]. Numerous studies have shown that the chemical toxicity of oil to marine organisms varies depending on its type and composition [2, 3]. In general, the toxicity of refined oil is higher than that of crude oil, and the toxicity of low molecular weight hydrocarbons is higher than that of high molecular weight hydrocarbons [4]. The toxicity of various hydrocarbons is generally in the order aromatic hydrocarbons > olefins > cyclohydrocarbons > chain hydrocarbons. Petroleum hydrocarbons are highly toxic to marine organisms, mainly because they disrupt the normal structure and permeability of cell membranes, interfere with the enzyme system of organisms, and thus affect their normal physiological and biochemical processes. Bioaccumulation in the food chain causes great harm to human health, including stomach diseases and even cancer. Petroleum hydrocarbons have been listed as key monitoring targets by the United Nations Environment Programme.

The commonly used methods for the degradation of marine petroleum hydrocarbon pollutants can be divided into physical, chemical, and biological methods [5, 6]. Physical methods are direct methods for oil repair and recovery that do not change the physical and chemical properties of the oil [7]. For example, adding 10 g/L commercial synthetic resin (XAD7) can remove 96.3% of emulsified oil in 25–50 mg/L simulated emulsified wastewater [8], and adding 20 g/L chitosan and acrylamide combined with a new hydrogel can remove 97% of 0.25 g/L crude oil wastewater [9]. These methods are simple and safe but have limitations, such as incomplete treatment and recycling, and are often suitable for controlling and recycling sudden oil spills. Chemical methods mainly involve burning the spilled oil onsite and adding materials to oil-contaminated water [10]. Chemical combustion can produce large amounts of particulate matter, CO_2_, and SO_2_ that can cause serious air pollution. Altaş et al [11] found that removal efficiency of chemical oxygen demand (COD) (50–80%) of petroleum refinery wastewater was obtained by using FeSO_4_·7H_2_O together with Ca(OH)_2_ as precipitant-aid. However, Fe^2+^ may be easily oxidized by oxygen in air, while Fe^3+^ easily forms hydroxide in water. In order to reduce the secondary pollution of the environment caused by the use of chemical agents during the oil removal process, environmentally friendly and renewable materials are being used. Peng et al. [12] summarized the uses of cellulose-based materials for crude oil spill cleaning. These materials have shown promising results in oil removal. However, the main issues such as the material preparation, used oil sorbents disposal still need to be further addressed.

Biological methods primarily involve bioremediation [13]. Currently, anaerobic degradation of pollutants in common bioremediation can be achieved through two pathways. One way is to generate energy by coupling substrate oxidation to respiration via the reduction of a non-oxygen terminal electron acceptor, and the other way is to generate energy via a fermentation pathway. These organisms, some bacteria (e.g., *Pseudomonas*, *Thiobacillus*, *Geobacter*) and some archaea (e.g., *Desulfobacterales*, *Methanosaeteta sp*., *Methanobacterium beijingense*), are called fermentative degraders [14]. Qaderi F et al. [15] built a moving-bed biofilm reactor (MBBR), in which a biofilm containing various microorganisms suitable for treating petroleum pollutants was prepared from the reflux sludge flow to the activated sludge unit at the Tehran refinery treatment plant. The results showed that retention time of 23 h, influent total petroleum hydrocarbon of 164.78 mg/L, and media filling ratio of 45% yielded, the highest efficiency of 97% in removing petroleum pollutants. A laboratory-scale study was conducted to evaluate the feasibility of a modified rotating biological contactor with polyurethane foam (RBC-PUF) attached to a disk as a porous support medium for the biodegradation of refinery wastewater. The RBC-PUF was inoculated with a seed culture obtained from an activated sludge unit treating the refinery wastewater, then a thin biofilm containing multiple microorganisms was formed for treating refinery wastewater. The results showed that the total COD and oil removal efficiency exceeded 87% and 80%, respectively [16]. Compared with traditional physicochemical methods, biological methods have the advantages of low cost, small environmental impact, and no secondary pollution, and can be used for fixed-point remediation, making them promising methods for petroleum hydrocarbon treatment [17].

Considering the complexity of petroleum hydrocarbon wastewater and the increasing concern about the toxicity of organic pollutants entering the environment, it is crucial to establish a predictive model that can quickly and accurately determine the biological toxicity of organic pollutants. Quantitative Structure Activity Relationship (QSAR) refers to the quantitative relationship between the molecular structure of organic pollutants and their biological toxicity [18]. QSAR is such an effective prediction model, and the development of the Toxicity Estimation Software Tool (TEST) conforms to the trend of QSAR research, including the development of large QSAR packages, mutual penetration with large databases, and the application of toxicity models, making this software of great significance in the field of toxicity assessment and successfully opening up a new path for QSAR research [19].

Therefore, this study aimed to investigate the effects of selected strains, strain dosage, petroleum hydrocarbon concentration, mixed strains, and mixing ratio on the degradation efficiency of petroleum hydrocarbons and explore the maximum degradation efficiency of petroleum hydrocarbons by strains. Exploring the biodegradation mechanisms provides a theoretical basis for improving the degradation efficiency of petroleum hydrocarbon wastewater. The results of this study will provide technical support and solutions for effective treatment of oil pollution. This technology can quickly and efficiently reduce the pollution caused by oil to the environment. Moreover, it can not only control point-source pollution and surface-source pollution, but also be extended to oil spill emergency response, thereby promoting the development of environmental protection.

## Materials and methods

### Experimental materials

The main reagents used in the experiment, LB medium, NaCl, and H_2_SO_4_, were purchased from Sinopharm Chemical Reagent Co., Ltd. (Beijing, China).

Commercial engine oil, used as simulated petroleum hydrocarbon wastewater, was obtained from an automobile maintenance company in Changzhou, China.

Two bacterial strains were isolated from the oil reservoir environment and named W01 and W02. Strains W01 and W02 were identified as *Stenotrophomonas acidaminiphila* (GenBank: EU352763.1) and *Ochrobactrum* (GenBank: KY471630.1), respectively. The methods for screening, isolation, and purification of strains can be found in S1 Section.

### Growth and degradation of petroleum hydrocarbons by strains W01 and W02

Individual cultivation of strains: Strains W01 and W02 were inoculated into 100 mL of sterilised LB liquid medium and activated in a shaking incubator (ZHLY-300S, Shanghai Zhichu Instrument Co., Ltd., China) for 20 h at 30°C and 140 rpm. The activated two strains were then inoculated into two 100 mL LB liquid culture media at a volume ratio of 2%, and incubated for 48 h at 30°C, 140 rpm, and salinity of 1% until the logarithmic phase of strain growth. Engine oil at a volume ratio of 2% was added to a conical flask containing the liquid culture medium. The above solution was cultured under shaking conditions at 30°C and 140 rpm for 5 days to investigate the degradation efficiency of the strain on oil.

Strain combination culture: Strains W01 and W02 were inoculated into 100 mL of sterilised LB liquid medium and activated for 20 h at 30°C and 140 rpm. The activated two strains were then inoculated into two 100 mL LB liquid culture media at a volume ratio of 2% and cultivated for 48 h under the conditions of 30°C, 140 rpm, and 1% salinity until the logarithmic phase of strain growth. W01 and W02 bacterial liquids were absorbed to form 100 mL of mixed bacterial liquid, with a combination ratio of W01: W02 = 1:1, W01: W02 = 2:1, and W01: W02 = 1:2, and 2% oil was added to the conical flask of liquid culture media. The above solution was cultured under shaking conditions at 30°C and 140 rpm for 5 days to investigate the degradation efficiency of the mixed strains on oil.

### Analytical methods

The analysis methods of total petroleum hydrocarbon (TPH) and hydrocarbon composition were same as described by Chang et al. [20]. Briefly, the TPH extractions were conducted in an automatic Soxhlet extractor (SOX 402 Micro, Gerhard Soxtherm, Germany) and analyzed using a gas chromatography-flame ionisation detector (GC-FID, 6890N, Agilent, USA). Then, the relative contents of the petroleum hydrocarbon components were analysed and determined using gas chromatography-mass spectrometry (GC-MS, 7890A-5975C, Agilent, USA). The testing method for GC-MS can be found in S2 Section. The toxicity of the intermediate products is the basis for determining whether the treatment method is feasible. Based on the median lethal dose (LD_50_) of oral rat as the toxicity parameter, the hierarchical clustering in TEST software, which is one of the QSAR model methods, was used to evaluate the oxicity of the products produced during the degradation of petroleum hydrocarbons [21]. The toxicity analysis method for intermediate products can be found in S3 Section.

## Results and discussion

### The effect of pure bacteria on the degradation of petroleum hydrocarbons

#### The effect of strain species on the degradation efficiency of petroleum hydrocarbons components

**Fig 1:**
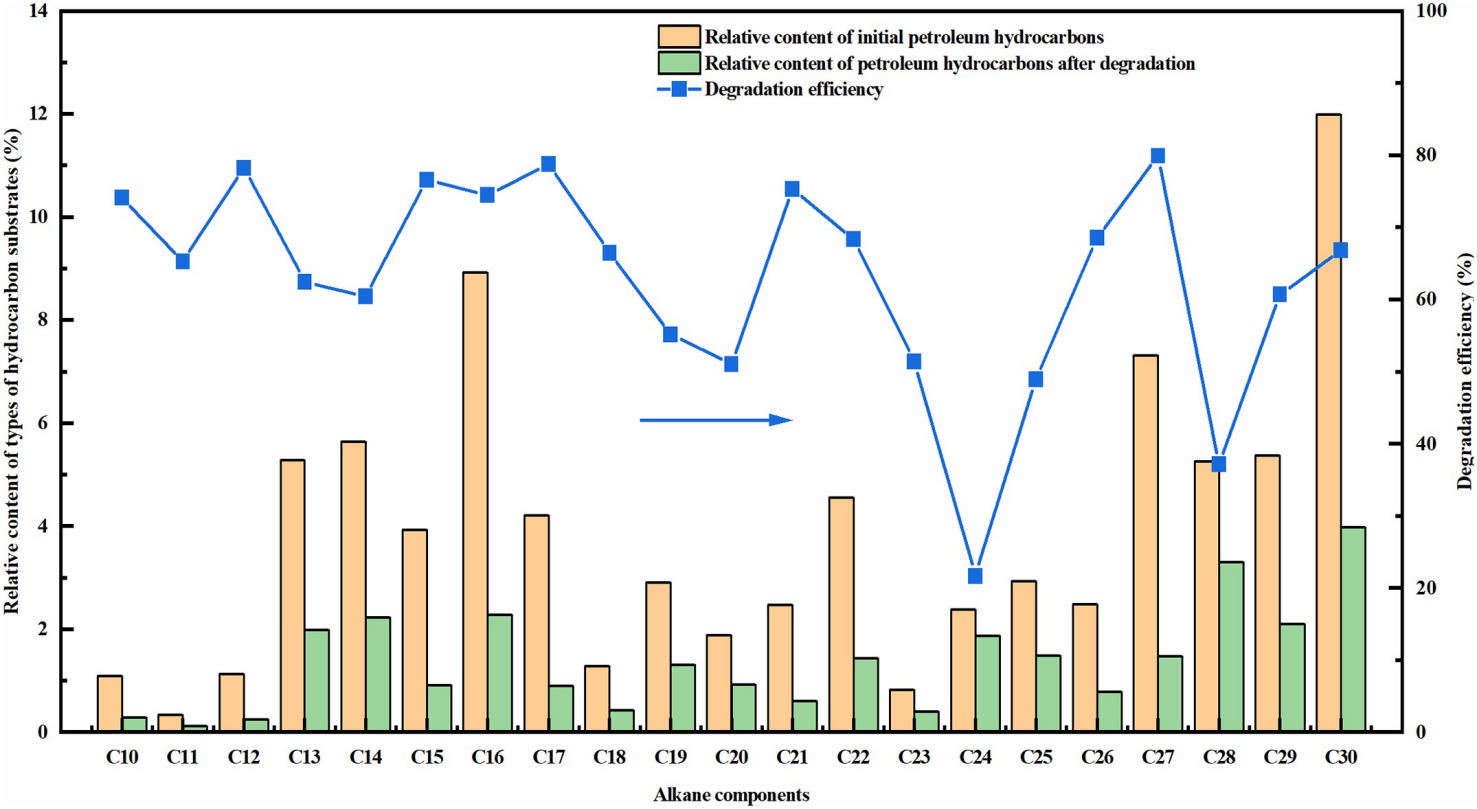
Degradation efficiency of strain W01 on the relative content of petroleum hydrocarbon components.

**Fig 2:**
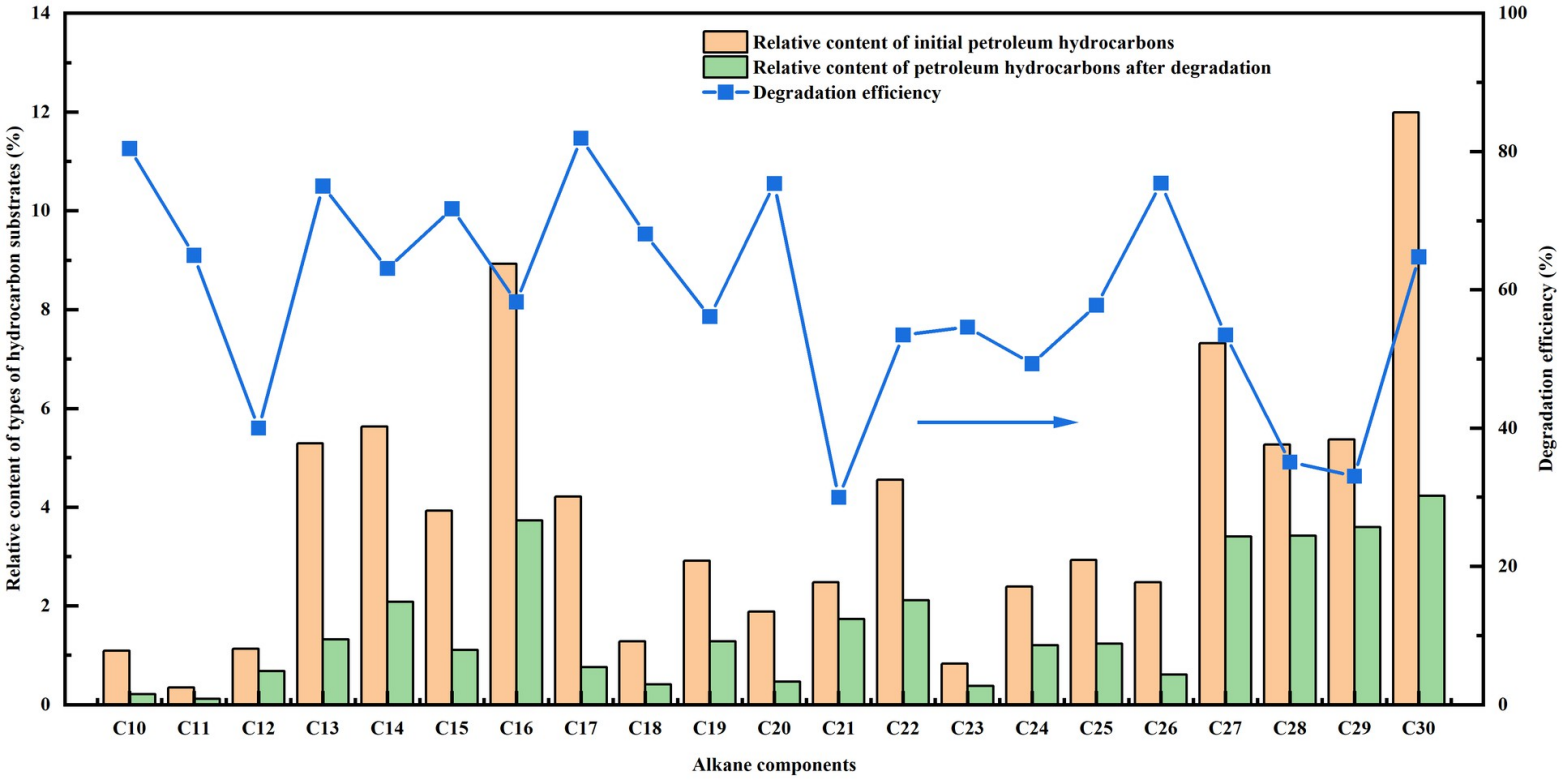
Degradation efficiency of strain W02 on the relative content of petroleum hydrocarbon components.

The effect of strain type on the degradation of petroleum hydrocarbons was studied. The degradation of petroleum hydrocarbons by strains W01 and W02 is shown in Figs 1 and 2.

As shown in Fig 1, although the strain W01 can consume both short- and medium-long carbon chain C10-C30 components in petroleum hydrocarbons, the degradation efficiencies of different petroleum hydrocarbon components by the strain are also different. The highest degradation efficiency of C27 by the strain W01 was 79.95%, whereas the lowest degradation effect was observed for C24, at only 21.71%. The average degradation efficiency of the strain W01 of petroleum hydrocarbon components was 62.98%. In the report of Gregorio et al. [22], the *Stenotrophomonas* sp. M1 strain was used to degrade diesel oil. After 6 days, the degradation efficiencies of C27 and C24 were 39.45% and 23.65%, respectively. C28, C29, and C30 achieved high degradation efficiencies of 46.17%, 45.99%, and 43.06%, respectively, while in this study, they were 37.15%, 60.74% and 66.86%, respectively, during 5 days operation (Table 1). Overall, the average degradation efficiency in this study was higher than that of Gregorio et al. [22], indicating that the strain W01 had a good degradation effect on petroleum hydrocarbons.

**Table 1:** The degradation effect of strains on petroleum hydrocarbons.

Bacterial strain	Degradation conditions	Degradation effect	References
***Stenotrophomonas***	Diesel oil concentration of 1%, treated at 28°C for 6 days	Degradation of C28 to C30 reaches 43%~46%	[22]
	Hexadecane concentration of 2.5%, treated at 30°C for 7 days	The degradation efficiency of total petroleum hydrocarbons reaches 74%	[29]
	Refinery wastewater, treated at 37°C for 21 days	The degradation efficiency of total petroleum hydrocarbons reaches 72.33%	[27]
	Petroleum refinery sludge of 2% (w/v), treated at 37°C for 28 days	The degradation of total petroleum hydrocarbon reaches 65 ± 2.4%	[25]
	Petroleum hydrocarbon concentration of 1 mL/L, treated at 30°C, 140 rpm for 5 days	The total degradation efficiency reaches 71.69%	This study
***Ochrobactrum***	Petroleum refinery wastewater, pH 7, treated at 150 rpm for 7 days	The total degradation efficiency reaches 53%	[28]
	Crude oil concentration of 4% (v/v), treated at 30°C for 3 weeks	The total degradation efficiency of the most hydrophobic components reaches 70%	[24]
	Lubricating Oil concentration of 4.6%, treated at 36.4°C, pH 7.3 for 7 days	The total degradation efficiency reaches 57%	[26]
	Crude oil fractions 500 N, lube oil, and de-asphalted oil at a concentration of 1 g/L, 37°C, 150 rpm treatment for 15 days	The petroleum hydrocarbons removal varied from 20%–30%.	[23]
	Petroleum hydrocarbon concentration of 1 mL/L, 30°C, 140 rpm treatment for 5 days	The total degradation efficiency reaches 78.34%	This study

As shown in Fig 2, the degradation effect of the strain W02 on different petroleum hydrocarbon components was also different. After 5 days, the strain W02 exhibited the best degradation efficiency of C17, reaching 81.93%, whereas the lowest degradation efficiency for C21 (29.97%). The average degradation efficiency of the strain W02 of petroleum hydrocarbon components was 59.14%. Kshirsagar et al. [23] found that in 15 days, *Ochrobactrum intermedium* could degrade hydrocarbons in crude oil fractions 500 N, lube oil, and de-asphalted oil. The hydrocarbons removal varied from 20%-30%. Thus, strain W02 also had the ability to degrade petroleum hydrocarbons.

### The effect of strain dosage on the degradation efficiency of petroleum hydrocarbons

**Fig 3:**
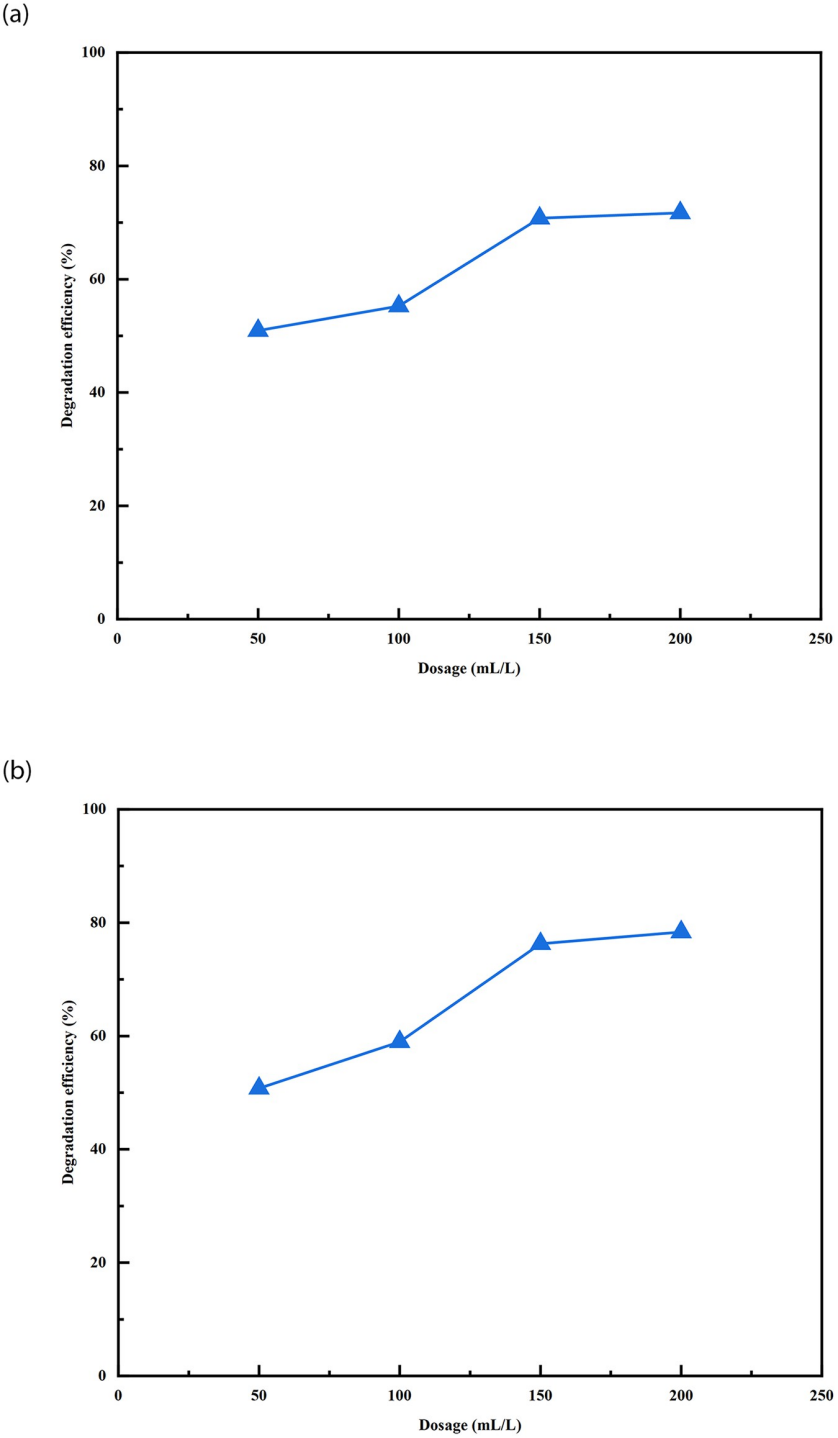
The influence of strain dosage on the degradation efficiency of total petroleum hydrocarbons (a: strain W01, b: strain W02).

To investigate the effect of different bacterial liquid dosages on the degradation of petroleum hydrocarbons under optimised conditions for the individual growth of the strains W01 and W02, 50, 100, 150, and 200 mL of the logarithmic growth phase bacterial liquid was added to 100 mL of wastewater with a petroleum hydrocarbon concentration of 1 mL/L while maintaining the same cultivation conditions. The above solution was cultured under shaking conditions at 30°C and 140 rpm for 5 days to investigate the degradation efficiency of TPH. The results are shown in Fig 3.

As shown in Fig 3, under the conditions of different dosages of 50, 100, 150, and 200 mL of bacterial solution, the degradation efficiencies of both strains for 1 mL/L of petroleum hydrocarbon wastewater showed a rapid initial increase, followed by a slow and stable trend. The degradation efficiency of the strain W01 on TPH was 50.93% at 50 mL and 55.26% at 100 mL. The degradation efficiency increased by 28.07% when the dosage increased to 150 mL, reaching 70.77%. Subsequently, it stabilised, and the degradation efficiency reached 71.69% at 200 mL.

The degradation efficiency of the strain W02 increased by 16.13% when the strain dosage was increased from 50 mL (50.77%) to 100 mL (58.96%). Then, the degradation efficiency of TPH increased by 29.38% when the dosage was increased from 100 mL to 150 mL, reaching 76.28%. Continue to increase the strain dosage to 200 mL, the degradation efficiency increased by 2.70%, reaching 78.34%.

It can be seen that the degradation efficiency of TPH increases as the amount of strain dosage increases. However, it is evident that as the strain dosage increases from 150 mL to 200 mL, the increase in degradation efficiency is not significant. This may be because as the amount of strains added increases, the increase in biosurfactant glycolipopeptidal will inhibit biodegradation, as previously studied [24]. Although the versatility of *Stenotrophomonas* and *Ochrobactrum* in degrading petroleum hydrocarbons is known [23–29], our findings further indicate that the isolate *Stenotrophomonas* and *Ochrobactrum* exhibited good engine oil degradation capacities. Notably, *Stenotrophomonas* and *Ochrobactrum* were responsible for degrading petroleum hydrocarbons, but it is difficult to have a comparative assessment since oil type and concentration varied across the studies (Table 1).

#### The effect of petroleum hydrocarbon concentration on bacterial degradation

**Fig 4:**
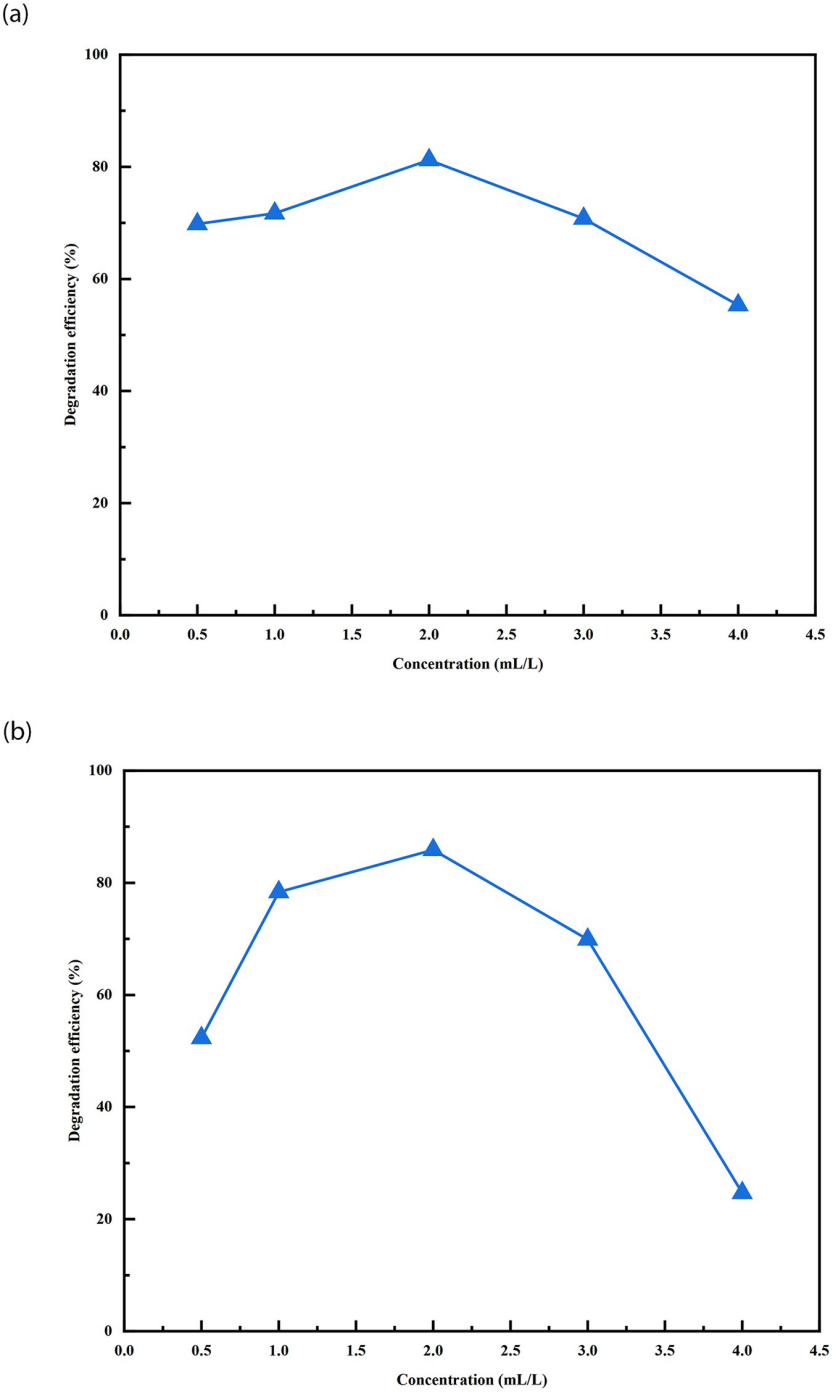
The influence of petroleum hydrocarbon concentration on the degradation efficiency of total petroleum hydrocarbons.

The 200 mL of wastewater with initial petroleum hydrocarbon concentrations were set to 0.5, 1, 2, 3, and 4 mL/L, while maintaining the same conditions as other cultures, to investigate the degradation of different petroleum hydrocarbon concentrations by the growth of the strains W01 and W02 under optimised conditions. The samples were incubated under shaking conditions at 30°C and 140 rpm for 5 days. The results are shown in Fig 4.

As shown in Fig 4, at initial petroleum hydrocarbon concentrations of 0.5, 1, 2, 3, and 4 mL/L, the degradation efficiency of TPH by both strains first increases and then decreases with an increase in the initial petroleum hydrocarbon concentration. Moreover, at a concentration of 2 mL/L, the degradation efficiency was optimal, reaching 81.22% and 85.88%. The reason for its decline may be due to the increase in petroleum hydrocarbon concentration inhibiting the destruction of the bacterial growth environment, thereby inhibiting the growth rate of bacteria and leading to a decrease in degradation efficiency, which has also been mentioned in a previous study [29].

### The effect of mixed bacteria on the degradation of petroleum hydrocarbons

**Fig 5:**
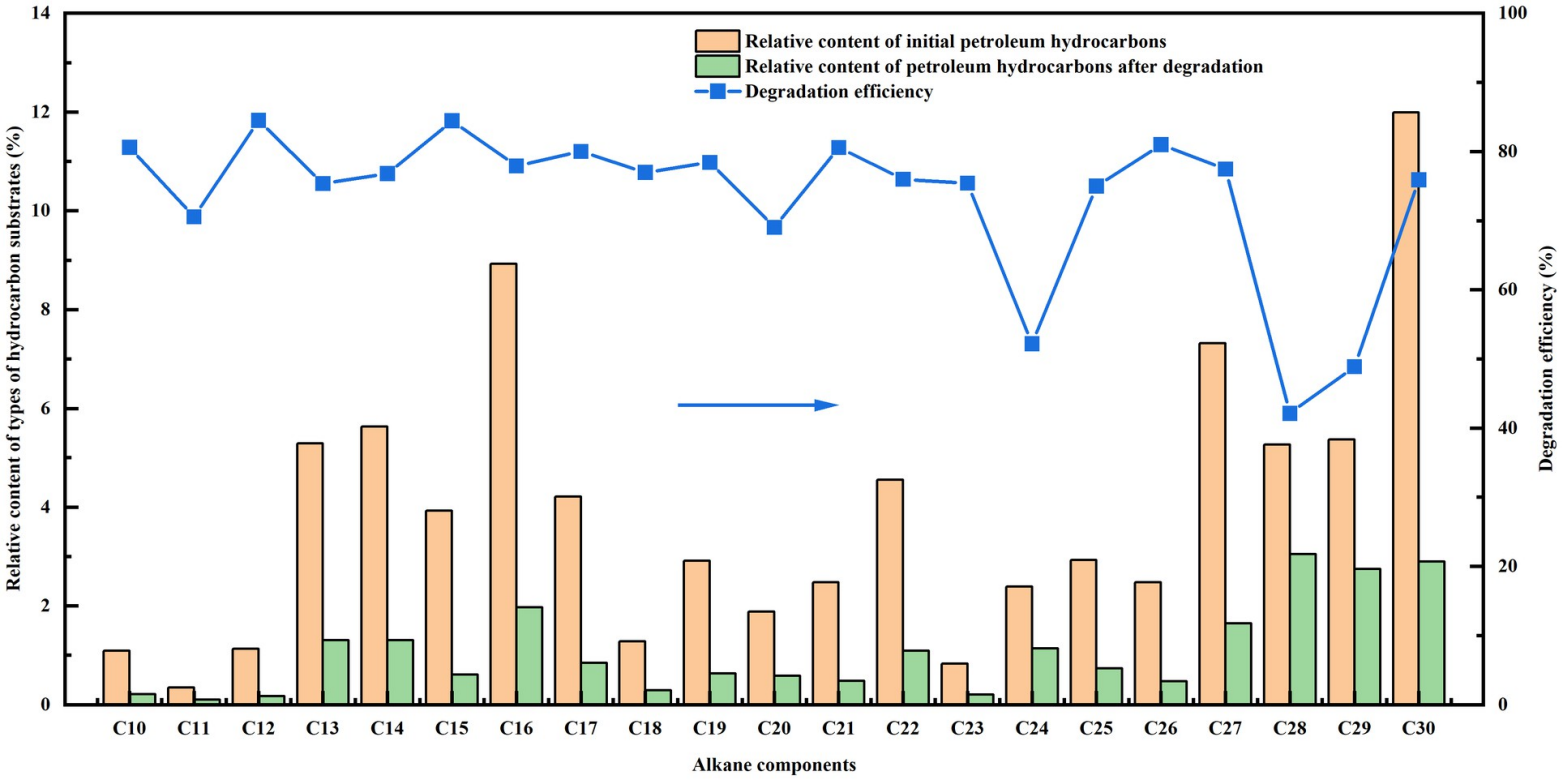
Degradation efficiency of mixed bacteria on the relative content of petroleum hydrocarbon components.

According to previous studies, there are differences in the consumption of petroleum hydrocarbons when W01 and W02 bacteria grow alone. Strains W01 and W02 were inoculated at a 1:1 ratio into 100 mL of wastewater with a petroleum hydrocarbon concentration of 1 mL/L and cultured for 5 days to investigate the effect of co-growth of the combined strains on the degradation of petroleum hydrocarbons. The results are shown in Fig 5.

Fig 5 shows that the combined strains can consume short- and medium-carbon chain C10-C30 components. The degradation efficiencies of C10, C12, C15, C17, C21, and C26 by mixed bacteria are all not below 80%. However, the degradation efficiencies of C10-C30 by strain W01 are all below 80%, while strain W02 only obtained degradation efficiencies above 80% for C10 and C17, respectively (as shown in Figs 1 and 2). The average degradation efficiency of the mixed strain of petroleum hydrocarbon components was 73.30%, which was 10.32% and 14.16% higher than that of strain W01 and W02 alone, respectively. This result was consistent with report of Kshirsagar et al. [23]. Kshirsagar et al. [23] discovered that mixing *Ochrobactrum intermedium* with other bacterial strains (*Pseudomonas putida*, *Pseudomonas mendocina*, *Bacillus cereus*, *Bacillus marisflavi*, *Lysinibacillus fusiformis*) could effectively degrade hydrocarbons in crude oil fractions 500 N, lube oil, and de-asphalted oil. The hydrocarbons removal varied from 40%-60%, which showed an increase of approximately 50% in biodegradation compared to *Ochrobactrum intermedium*. The levels of the C21-C30, C31-C35, and C36-C60 decreased by 13%–54%, 49%–70%, and 39%–60%, respectively.

**Fig 6:**
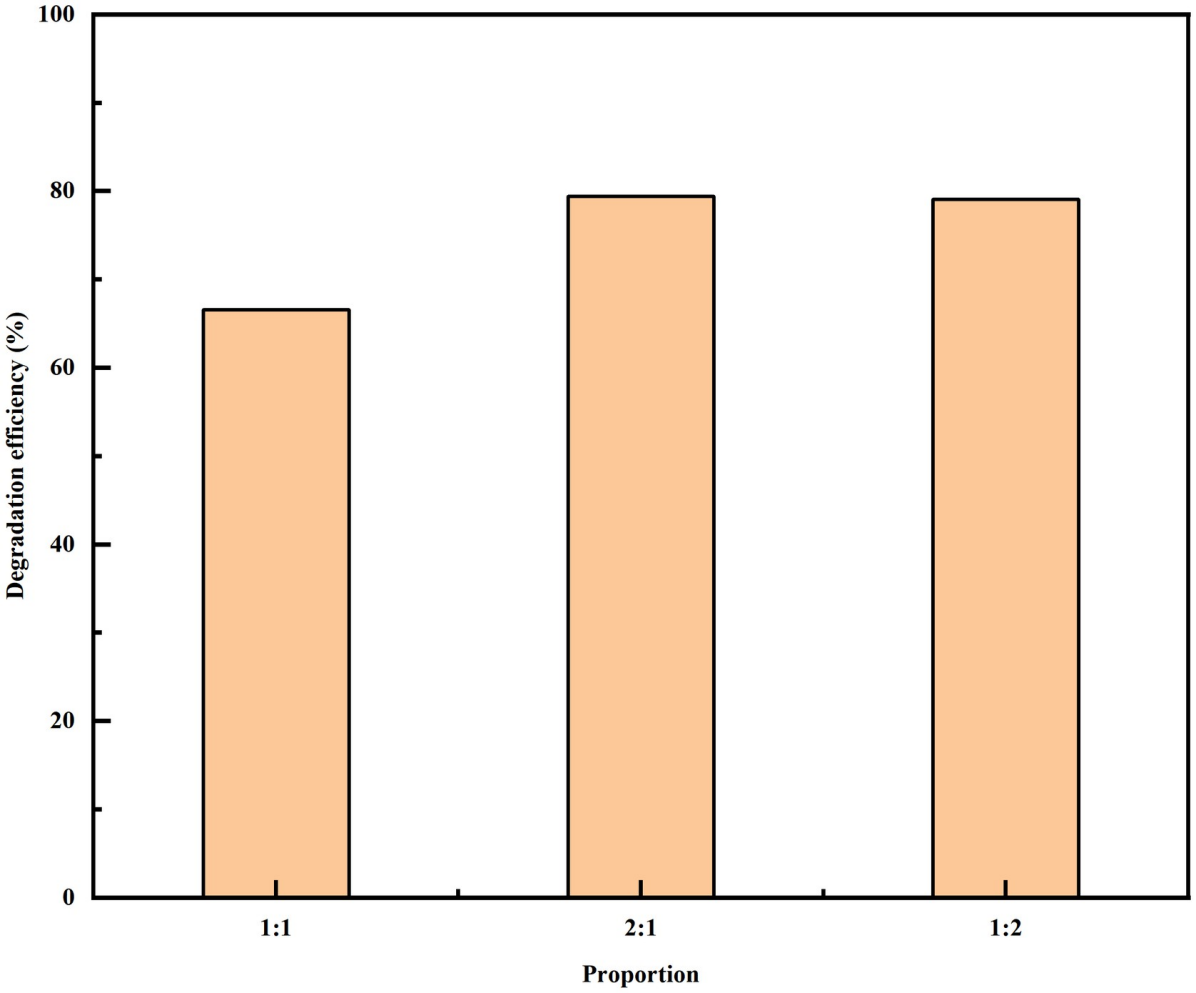
The influence of strains mixing ratio on the degradation efficiency of total petroleum hydrocarbons.

To investigate the consumption of petroleum hydrocarbons by the growth of the two bacterial strains in different proportions under optimised conditions, the two strains were mixed and cultured in different combinations according to W01: W02 = 1:1/1:2/2:1. The results are presented in Fig 6.

Fig 6 shows that, compared to the 1:1 ratio, when the mixing ratios are 1:2 and 2:1, the combined strain has a higher degradation efficiency of TPH during growth, which is approximately 12.0% higher. However, the degradation efficiency of different mixing ratios can be maintained above 60%, which also indicates that the consumption of petroleum hydrocarbons by the mixed strains under the same growth conditions is stronger than that of W01 and W02 bacteria growing alone. Similar situations have also occurred in previous study [22]. Teng et al. [30] constructed a mixed cold-adapted bacteria flora, which contained *Stenotrophomonas* sp., to treat contaminated water (1 g oil/L). The mixed flora demonstrated 53.68% of TPH removal after 30 days of incubation under 10°C. This may be because the characteristic of oil pollution is the presence of saturated components composed of alkanes and more stubborn unsaturated components. In this case, a combination of multiple bacteria has a wider and different enzymatic ability, thereby improving the degradation level of pollution [31]. Moreover, different strains of bacteria grow and reproduce in the same environment, competing for nutrients and substrates. At the same time, their own metabolites and other factors can have a comprehensive impact on microbial growth [32].

### Analysis of the degradation mechanism of petroleum hydrocarbons

#### GC-MS analysis of metabolic products in the degradation of petroleum hydrocarbons

Four intermediates, namely n-eicosane, 1-neneneba hexadecane alcohol, 2-octyldodecanol, and 2,4-di-tert-butylphenol, were detected using GC-MS during the degradation of petroleum hydrocarbons by strain W01 (Table 2 and S1–S4 Figs).

**Table 2:** GC-MS analysis of the metabolites during the degradation of petroleum hydrocarbons.

Material peak	residence time	Mass to charge ratio of ion fragments m/z	Corresponding substance
**I**	18.944	57 (M^+^)	n-eicosane
**II**	16.022	55 (M^+^)	1-neneneba hexadecane alcohol
**III**	17.673	57 (M^+^)	2-Octyldodecanol
**IV**	13.830	191 (M^+^)	2,4-di-tert-butylphenol

During petroleum hydrocarbon degradation by the strain W02, four intermediates were detected using GC-MS, namely diphenylamine, lauryl methacrylate, 2-Ethylhexanol, and ethyl hexyl benzoate (Table 3 and S5–S8 Figs).

**Table 3:** GC-MS analysis of the metabolites during the degradation of petroleum hydrocarbons.

Material peak	residence time	Mass to charge ratio of ion fragments m/z	Corresponding substance
**I**	14.913	169 (M^+^)	diphenylamine
**II**	15.918	69 (M^+^)	lauryl methacrylate
**III**	8.344	57 (M^+^)	2-Ethylhexanol
**IV**	15.458	105 (M^+^)	ethyl hexyl benzoate

#### Metabolomics analysis of differentially expressed genes

The correlation of differentially expressed genes between the two samples was tested using the original number of reads. According to the correlation heat map of the samples in S9 Fig, the correlation between the samples is strong. The Pearson product-moment correlation coefficient between the two biological replicates of the sample was > 0.9, and the differential gene expression levels between the sample and blank groups tended to be the same. According to the heat map analysis, the biological repeatability of sequencing was good, and the data were reliable.

S10 and S11 Figs showed that there are 397 differentially expressed genes between the strain W01 sample and the blank sample, of which 44 are significantly upregulated, such as N-Butyryl-L-homoserine lactone, Glycyl-leucine, and 73 are significantly downregulated, such as eryyhro-3-Hydroxy-Ls-aspartate, 2-Deoxyguanosine, Propazine, Dimethylglycine. There were 397 differentially expressed genes between the W02 and blank samples, of which 32 are significantly upregulated, such as Procollagen 5-hydroxy-L-lysine, Propazine, N6-Acetyl-N6-hydroxy-L-lysine, Betaine, Pantothenic acid, and 27 are significantly downregulated, such as Ureidopropionic acid, 4-Hydroxycinnamic acid, Pantothenic acid, (R)-3-Hydroxybutyric acid, Deoxyinosine.

**Fig 7:**
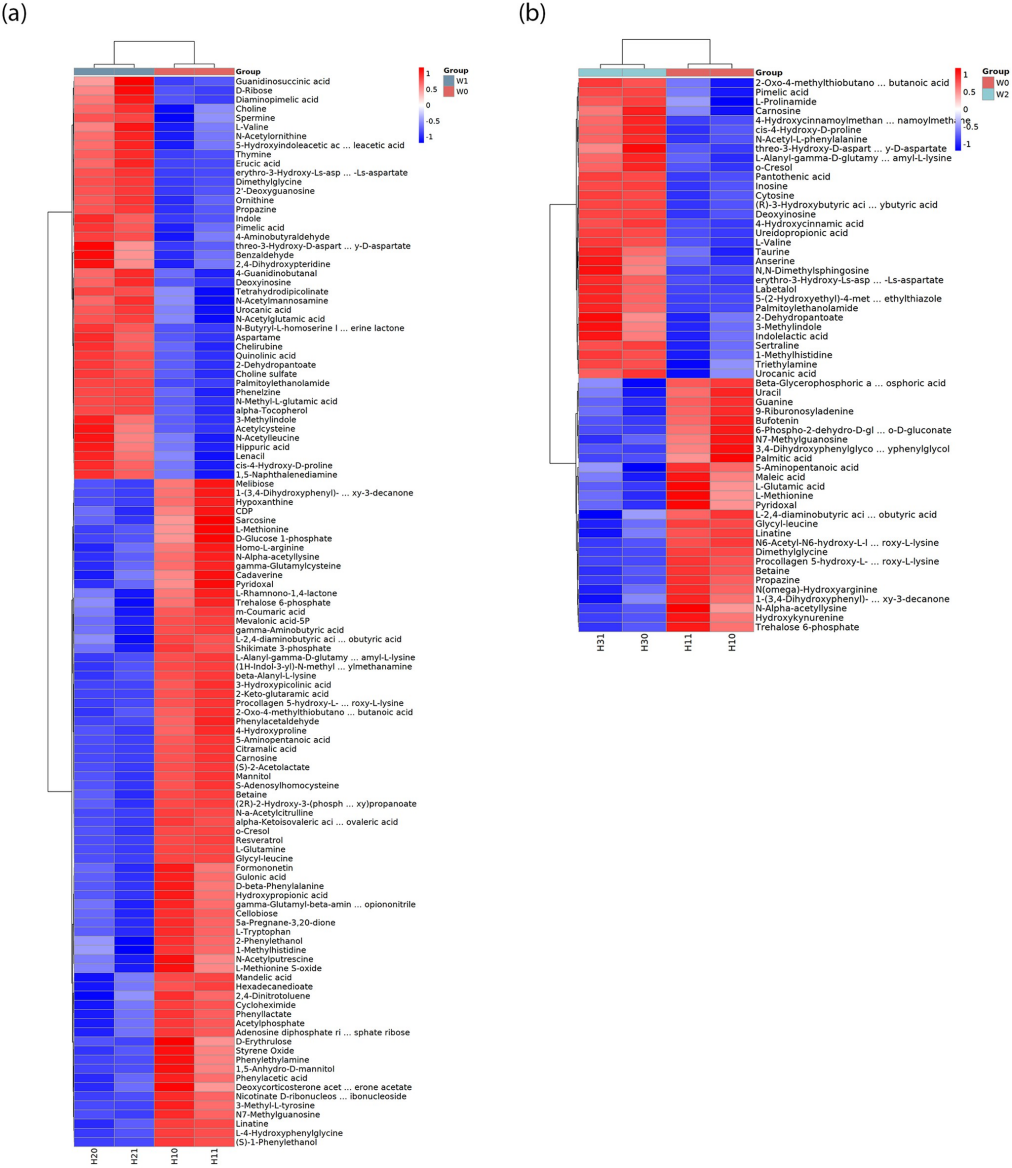
The Differential molecular heatmaps (a: strain W01, b: strain W02).

Fig 7(a) shows the differential expression heatmaps of the strain W01 and the blank control group. The treated samples showed the following substances: L-Alanyl-gamma-D-glutamyl-L lysine (C14), 1- (3,4-Dihydroxyphenyl) -5-hydroxy-3-decanone (C16), Reversrol (C14), and other components. Simultaneously, the differential expression heatmaps of the strain W02 and the blank control group were analysed. As shown in Fig 7(b), the result also indicated the presence of the following substances: L-Alanyl-gamma-D-glutamyl-L-lysine (C14), Lenacil (C13), 1- (3,4-Dihydroxyphenyl) -5-hydroxy-3-decane1 (C16), Labelalol (C19), and other components, which were mutually confirmed from the intermediate products detected using GC-MS. According to Sajna et al. [33] and Zhang et al. [34], benzoate and phenylacetic acid are the upstream and downstream products of catechol, respectively, and heptylcyclohexane is broken into alkanes by intermediate enzymes or bases.

#### Preliminary derivation of degradation pathways

As strains that can grow and reproduce using petroleum hydrocarbons as the sole carbon source, the mechanisms by which strains W01 and W02 degrade petroleum hydrocarbons have become important research targets, and multiple scholars have explored their degradation mechanisms [35, 36].

According to the GC-MS detection and metabolomic determination, n-eicosane was present in the wastewater before strain W01 degradation, whereas n-eicosane was not detected in the wastewater after degradation, and the two intermediate products, 1-nenenebc hexadecane alcohol and 2-octyldodecanol, were obtained. Diphenylamine and lauryl methacrylate were present in the wastewater before the strain W02 degradation, whereas diphenylamine and lauryl methacrylate were not detected in the wastewater after degradation, and 2-Ethylhexanol and ethyl hexyl benzoate were obtained.

**Fig 8:**
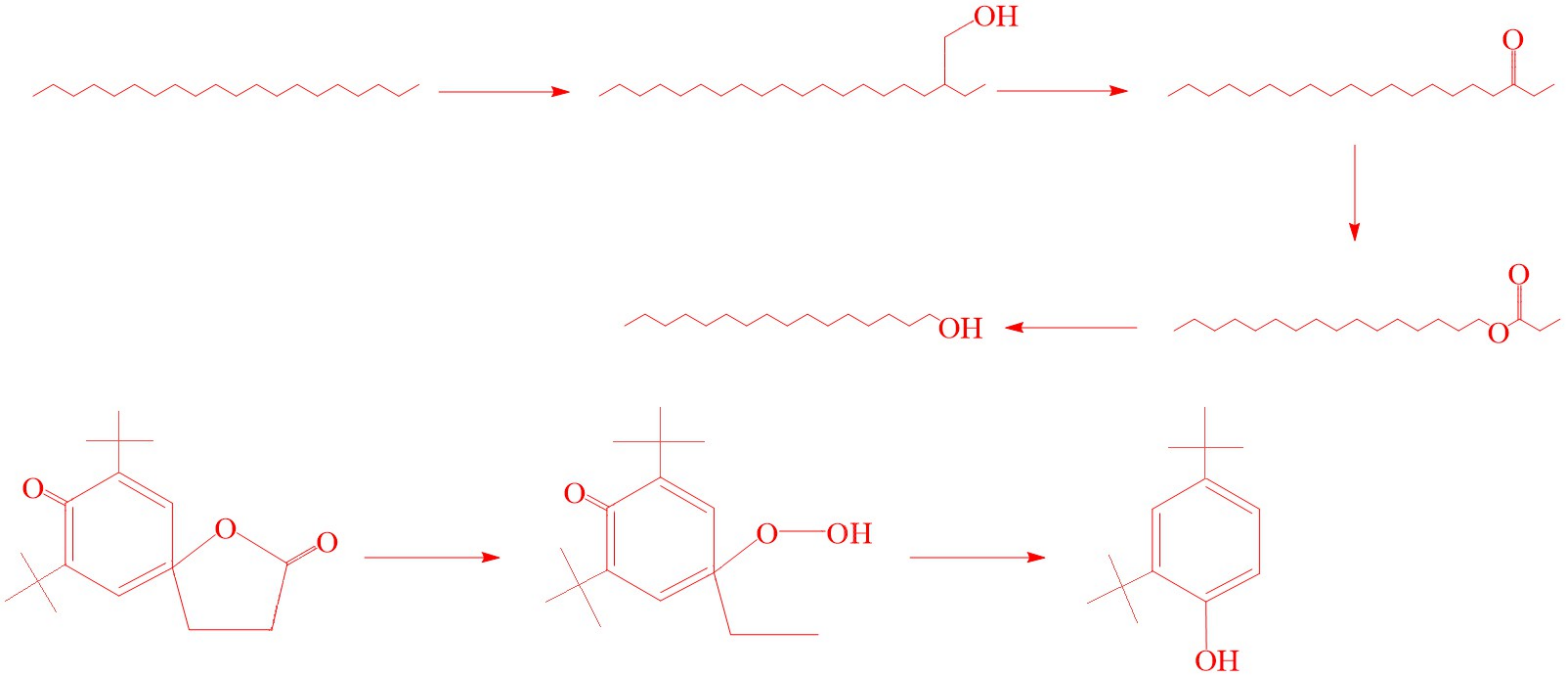
Preliminary inference on the degradation pathway of petroleum hydrocarbons by strain W01.

**Fig 9:**
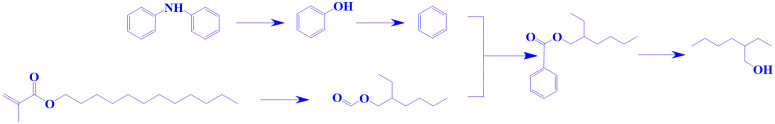
Preliminary inference on the degradation pathway of petroleum hydrocarbons by strain W02.

In the degradation of petroleum hydrocarbons by the strain W01, n-eicosane was first oxidized to 2-octyldodecanol, the hydroxyl group of 2-octyldodecanol was further oxidized to the aldehyde group, the aldehyde group was further oxidized to the ester group, and finally the carbon oxygen bond broke to produce 1-nenenebb hexadecane alcohol, and the C-O bond in 7,9-di-tert-butyl-1-oxa [4.5] deca-6,9-dien-2,8-dione cracked to carboxylate, and finally degraded to 2,4-di-tert-butylphenol (Fig 8) [37–39]. In the degradation of petroleum hydrocarbons by the strain W02, diphenylamine may react with hydroxyl radicals to generate benzene; the C-C bond of lauryl methacrylate broke, and benzene generated ethyl hexyl benzoate, followed by the C-O bond breaking to generate 2-Ethylhexanol (Fig 9) [40]. However, owing to the complexity and variability of microbial degradation processes, and the considerable impact of environmental factors, further refinement of degradation pathways requires more in-depth research.

### Toxicity assessment of intermediate products in petroleum hydrocarbon wastewater degradation by strains

Table 4 presents the predicted results for the intermediate product LD_**50**_ detected in the W01 and W02 bacterial petroleum hydrocarbon-degrading wastewater systems. In the strain W01-degrading petroleum hydrocarbon wastewater system, the decrease in the content of n-eicosane indicates that it can be degraded by strain W01 into small molecule substances. Meanwhile, LD_50_ of intermediate products such as 2-octyldodecanol and 2,4-di-tert-butylphenol, is significantly lower than that of n-eicosane. Besides, although the LD_50_ value of 1-neneneba hexadecane alcohol is high, its content in the degraded wastewater is much lower than other substances. In the strain W02-degrading petroleum hydrocarbon wastewater system, the LD_50_ values of the two newly generated substances, 2-Ethylhexanol and ethyl hexyl benzoate, inferred through degradation pathways significantly lower than that of lauryl methacrylate in the original petroleum hydrocarbon wastewater. This indicates a decreasing trend in the toxicity of the reaction system. Therefore, from the perspective of toxicity assessment, strains W01 and W02 have great potential for remediating petroleum hydrocarbon wastewater.

**Table 4:** Prediction results of LD_50_, an intermediate product of petroleum hydrocarbon wastewater degradation by strains.

Chemical Name	CAS	Rat oral LD_50_ (mg/kg)
**n-eicosane**	112-95-8	7058.08
**1-neneneba hexadecane alcohol**	34513-50-3	11577.27
**2-octyldodecanol**	36653-82-4	4149.39
**2,4-di-tert-butylphenol**	96-76-4	1174.50
**diphenylamine**	122-39-4	2327.45
**lauryl methacrylate**	142-90-5	22944.96
**2-Ethylhexanol**	104-76-7	2728.13
**ethyl hexyl benzoate**	5444-75-7	6773.63

## Conclusion

Two strains, W01 and W02, were cultured and identified as *Stenotrophomonas acidaminiphila* and *Ochrobactrum*, respectively. The degradation ability of the two strains to petroleum hydrocarbons was studied by changing the strain type, strain dosage, petroleum hydrocarbon concentration, mixed strains, and mixed ratio on the degradation effect of petroleum hydrocarbons. The results showed that the degradation efficiency of petroleum hydrocarbons by the mixed strains was higher than that when the strains grew alone. When the ratio was 1:2 or 2:1, the degradation efficiency of petroleum hydrocarbons by the mixed strains during growth was relatively high, reaching 80%.

Intermediate products were identified through GC-MS and differential expression gene metabolomics analyses, and potential degradation pathways for petroleum hydrocarbon wastewater were proposed. In the degradation of petroleum hydrocarbons by the strain W01, n-eicosane was first oxidized to 2-octyldodecanol, the hydroxyl group of 2-octyldodecanol was further oxidized to the aldehyde group, the aldehyde group was further oxidized to the ester group, and finally the carbon oxygen bond broke to produce 1-nenenebb hexadecane alcohol, and the C-O bond in 7,9-di-tert-butyl-1-oxa [4.5] deca-6,9-dien-2,8-dione was cracked and then carboxylated, and finally degraded to 2,4-di-tert-butylphenol. In the degradation of petroleum hydrocarbons by the strain W02, diphenylamine may react with hydroxyl radicals to generate benzene; the C-C bond of lauryl methacrylate broke with benzene to generate ethyl hexyl benzoate, and the C-O bond broke to generate 2-Ethylhexanol.

The toxicity assessment of the intermediate products showed a substantial decrease in the LD_50_ of the final intermediate products, demonstrating the potential of W01 and W02 for degrading petroleum hydrocarbons.

## Supporting information

S1 Fig Mass spectrum of n-eicosane. (DOCX)

S2 Fig Mass spectrum of 1-neneneba hexadecane alcohol. (DOCX)

S3 Fig Mass spectrum of 2-octyldodecanol. (DOCX)

S4 Fig Mass spectrum of 2,4-ditert-butylphenol. (DOCX)

S5 Fig Mass spectrum of diphenylamine. (DOCX)

S6 Fig Mass spectrum of lauryl methacrylate. (DOCX)

S7 Fig Mass spectrum of 2-Ethylhexanol. (DOCX)

S8 Fig Mass spectrum of ethyl hexyl benzoate. (DOCX)

S9 Fig Heatmap of the correlation of mRNA expression between the samples (a: strain W01, b: strain W02). (DOCX)

S10 Fig Volcano plot of gene expression (a: strain W01, b: strain W02). (DOCX)

S11 Fig Statistic of differently expressed metabolite. (DOCX)

S1 Section Screening, isolation, and purification of bacterial strains. (DOCX)

S2 Section The testing method of GC-MS. (DOCX)

S3 Section The toxicity analysis method for intermediate products. (DOCX)

S1 Data Datasets used in the research—Compressed files archive. (ZIP)
