# Supplementary material for: Two types of microorganisms isolated from petroleum hydrocarbon pollutants: Degradation characteristics and metabolic pathways analysis of petroleum hydrocarbons
Source: PLoS One. 2024 Nov 13;19(11):e0312416. doi: 10.1371/journal.pone.0312416 (PMC11559972; doi:10.1371/journal.pone.0312416)
Supplement: S1 Fig — (DOCX) [file pone.0312416.s001.docx]

**S1 Fig. Mass spectrum of n-eicosane**


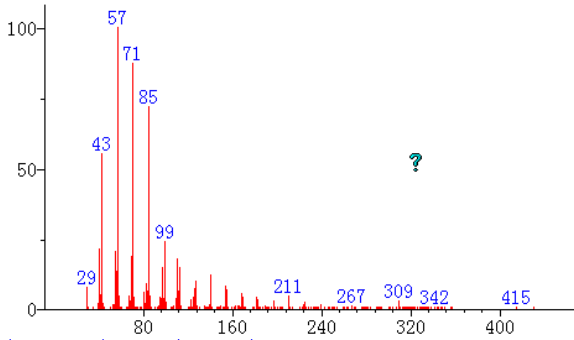

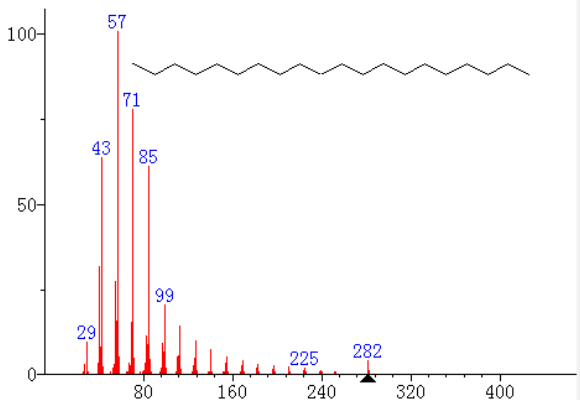


Fig.S1 shows the mass spectrum of substance peak I, with a residence time of 18.944 minutes and a mother ion m/z of 57 (M+). Comparing the mass spectrum of peak I with the standard n-eicosane, it was found that the two were similar. Therefore, substance I was determined to be n-eicosane.
