# Supplementary material for: Two types of microorganisms isolated from petroleum hydrocarbon pollutants: Degradation characteristics and metabolic pathways analysis of petroleum hydrocarbons
Source: PLoS One. 2024 Nov 13;19(11):e0312416. doi: 10.1371/journal.pone.0312416 (PMC11559972; doi:10.1371/journal.pone.0312416)
Supplement: S2 Fig — (DOCX) [file pone.0312416.s002.docx]

**S2 Fig. Mass spectrum of 1-neneneba hexadecane alcohol**


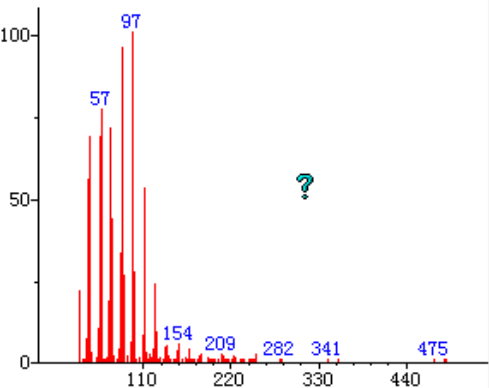

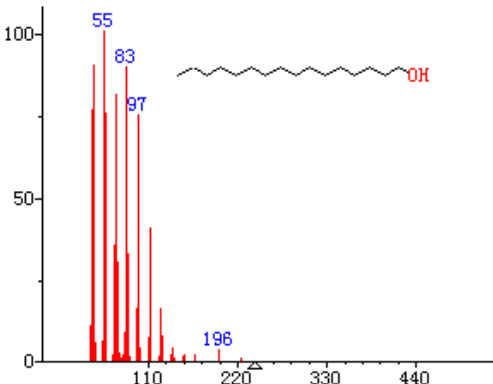


Fig.S2 shows the mass spectrum of the substance peak Ⅱ, whose residence time is 16.022 min, and the parent ion m/z is 55 (M+). Comparing the mass spectrum of the peak Ⅱ with that of the standard 1-neneneba hexadecane alcohol, it is found that the two are similar, so the substance Ⅱ is determined to be 1-nenenebb hexadecane alcohol.
