# Supplementary material for: Two types of microorganisms isolated from petroleum hydrocarbon pollutants: Degradation characteristics and metabolic pathways analysis of petroleum hydrocarbons
Source: PLoS One. 2024 Nov 13;19(11):e0312416. doi: 10.1371/journal.pone.0312416 (PMC11559972; doi:10.1371/journal.pone.0312416)
Supplement: S3 Fig — (DOCX) [file pone.0312416.s003.docx]

**S3 Fig. Mass spectrum of 2-octyldodecanol**


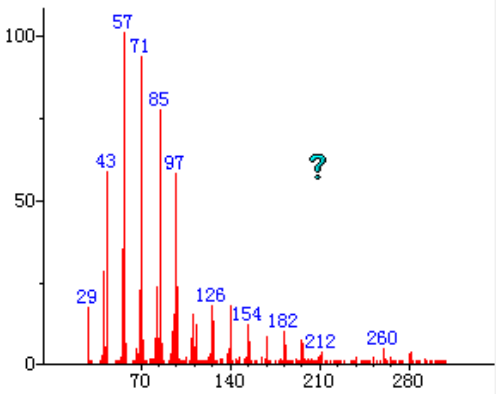

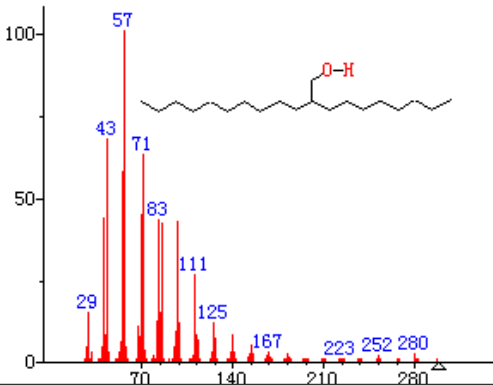


Fig.S3 shows the mass spectrum of substance peak Ⅲ, with a residence time of 17.673 minutes and a mother ion m/z of 57 (M+). Comparing the mass spectrum of peak Ⅲ with the standard 2-octyldodecanol, it was found that the two were similar. Therefore, substance Ⅲ was determined to be 2-octyldodecanol.
