# Supplementary material for: Two types of microorganisms isolated from petroleum hydrocarbon pollutants: Degradation characteristics and metabolic pathways analysis of petroleum hydrocarbons
Source: PLoS One. 2024 Nov 13;19(11):e0312416. doi: 10.1371/journal.pone.0312416 (PMC11559972; doi:10.1371/journal.pone.0312416)
Supplement: S4 Fig — (DOCX) [file pone.0312416.s004.docx]

**S4 Fig. Mass spectrum of 2,4-ditert-butylphenol**


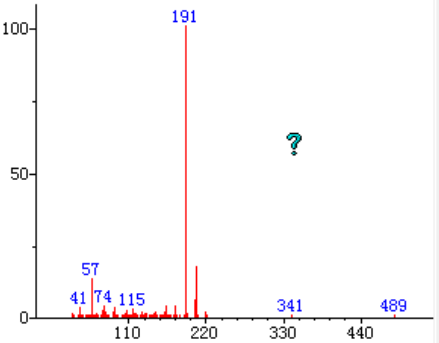

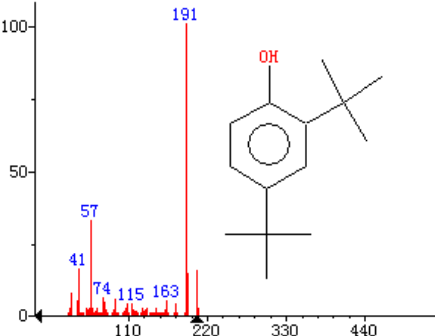


Fig.S4 shows the mass spectrum of the substance peak Ⅳ, with a residence time of 13.830 minutes and a mother ion m/z of 191 (M+). Comparing the mass spectrum of peak Ⅳ with the standard 2,4-ditert-butylphenol, it was found that the two were similar. Therefore, the substance Ⅳ was determined to be 2,4-ditert-butylphenol.
