# Supplementary material for: Two types of microorganisms isolated from petroleum hydrocarbon pollutants: Degradation characteristics and metabolic pathways analysis of petroleum hydrocarbons
Source: PLoS One. 2024 Nov 13;19(11):e0312416. doi: 10.1371/journal.pone.0312416 (PMC11559972; doi:10.1371/journal.pone.0312416)
Supplement: S5 Fig — (DOCX) [file pone.0312416.s005.docx]

**S5 Fig. Mass spectrum of diphenylamine**


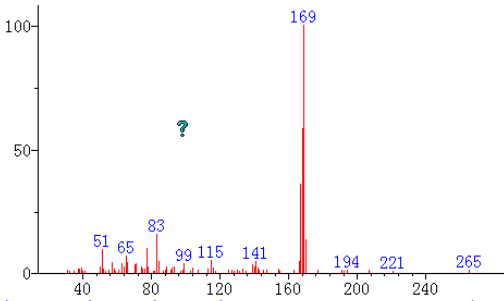

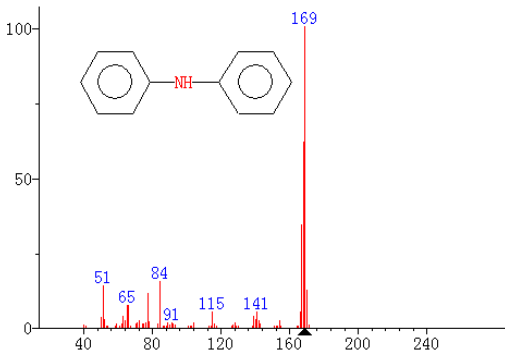


Fig.S5 shows the mass spectrum of the substance peak I, whose residence time is 14.913 min, and the parent ion m/z is 169 (M+). Comparing the mass spectrum of the peak I with that of the standard diphenylamine, it is found that the two are similar, so it is preliminarily inferred that the substance I is diphenylamine.
