# Supplementary material for: Two types of microorganisms isolated from petroleum hydrocarbon pollutants: Degradation characteristics and metabolic pathways analysis of petroleum hydrocarbons
Source: PLoS One. 2024 Nov 13;19(11):e0312416. doi: 10.1371/journal.pone.0312416 (PMC11559972; doi:10.1371/journal.pone.0312416)
Supplement: S6 Fig — (DOCX) [file pone.0312416.s006.docx]

**S6 Fig. Mass spectrum of lauryl methacrylate**


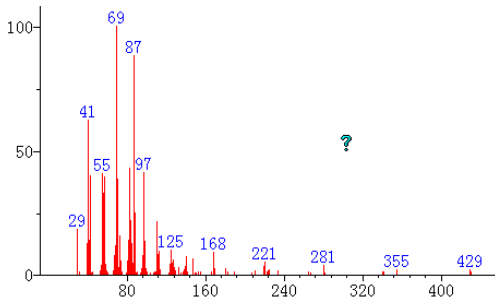

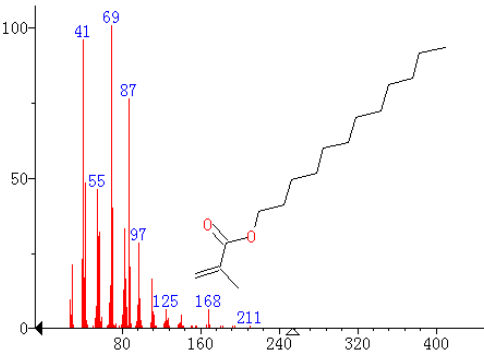


Fig.S6 shows the mass spectrum of substance peak II, with a residence time of 15.918 minutes and a mother ion m/z of 69 (M+). Comparing the mass spectrum of peak II with the standard product lauryl methacrylate, it was found that the two were similar, so substance II was determined to be lauryl methacrylate.
