# Supplementary material for: Two types of microorganisms isolated from petroleum hydrocarbon pollutants: Degradation characteristics and metabolic pathways analysis of petroleum hydrocarbons
Source: PLoS One. 2024 Nov 13;19(11):e0312416. doi: 10.1371/journal.pone.0312416 (PMC11559972; doi:10.1371/journal.pone.0312416)
Supplement: S7 Fig — (DOCX) [file pone.0312416.s007.docx]

**S7 Fig. Mass spectrum of 2-Ethylhexanol**


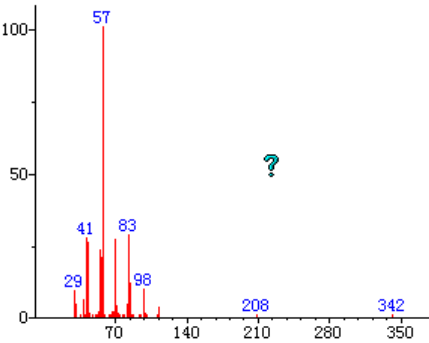

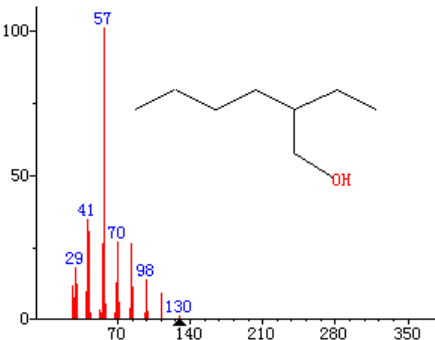


Fig.S7 shows the mass spectrum of the substance peak III, whose retention time is 8.344 min, and the parent ion m/z is 57 (M+). Comparing the mass spectrum of the peak III with that of the standard 2-Ethylhexanol, it is found that the two are similar, so it is preliminarily inferred that the substance III is 2-Ethylhexanol.
