# Supplementary material for: Two types of microorganisms isolated from petroleum hydrocarbon pollutants: Degradation characteristics and metabolic pathways analysis of petroleum hydrocarbons
Source: PLoS One. 2024 Nov 13;19(11):e0312416. doi: 10.1371/journal.pone.0312416 (PMC11559972; doi:10.1371/journal.pone.0312416)
Supplement: S8 Fig — (DOCX) [file pone.0312416.s008.docx]

**S8 Fig. Mass spectrum of ethyl hexyl benzoate**


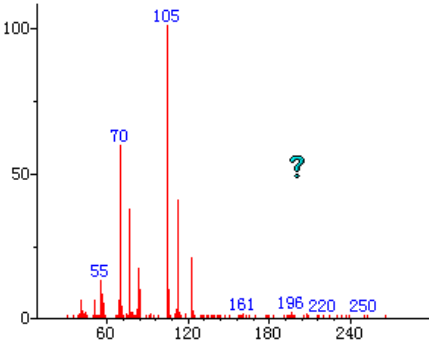

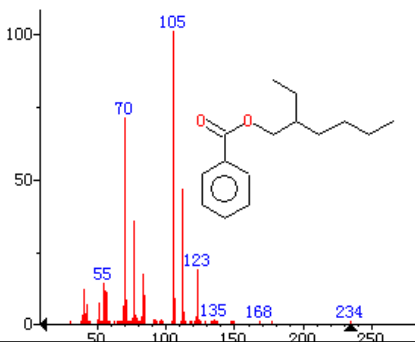


Fig.S8 shows the mass spectrum of substance peak IV, with a residence time of 15.458 minutes and a mother ion m/z of 105 (M+). Comparing the mass spectrum of substance peak IV with the standard substance ethyl hexyl benzoate, it was found that the two were similar, so it is preliminarily inferred that substance IV is ethyl hexyl benzoate.
