# Supplementary figures and images for: Two types of microorganisms isolated from petroleum hydrocarbon pollutants: Degradation characteristics and metabolic pathways analysis of petroleum hydrocarbons
Source: PLoS One. 2024 Nov 13;19(11):e0312416. doi: 10.1371/journal.pone.0312416 (PMC11559972; doi:10.1371/journal.pone.0312416)

**S9 Fig. Heatmap of the correlation of mRNA expression between the samples (a: strain W01, b: strain W02)**


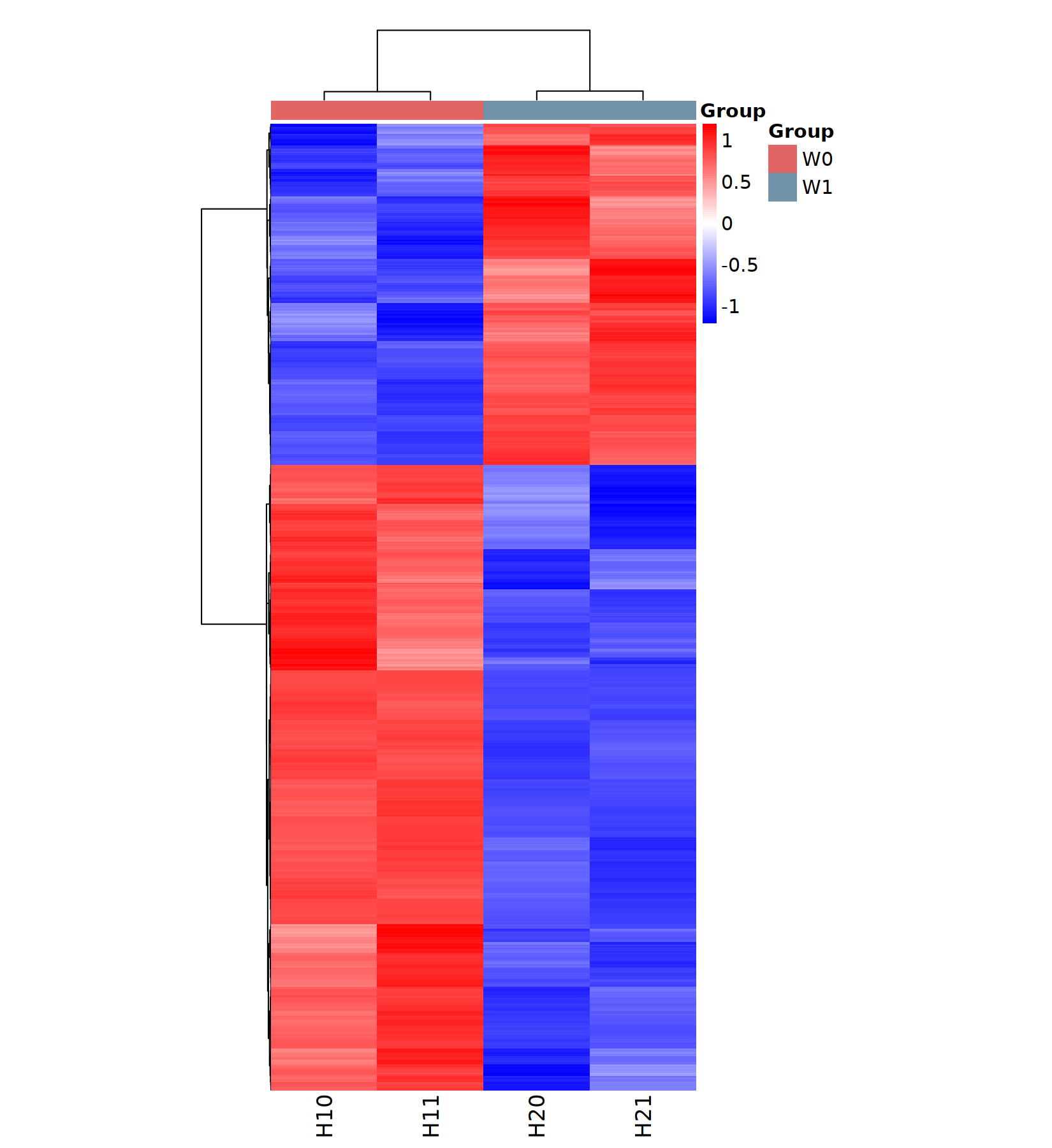


(a)


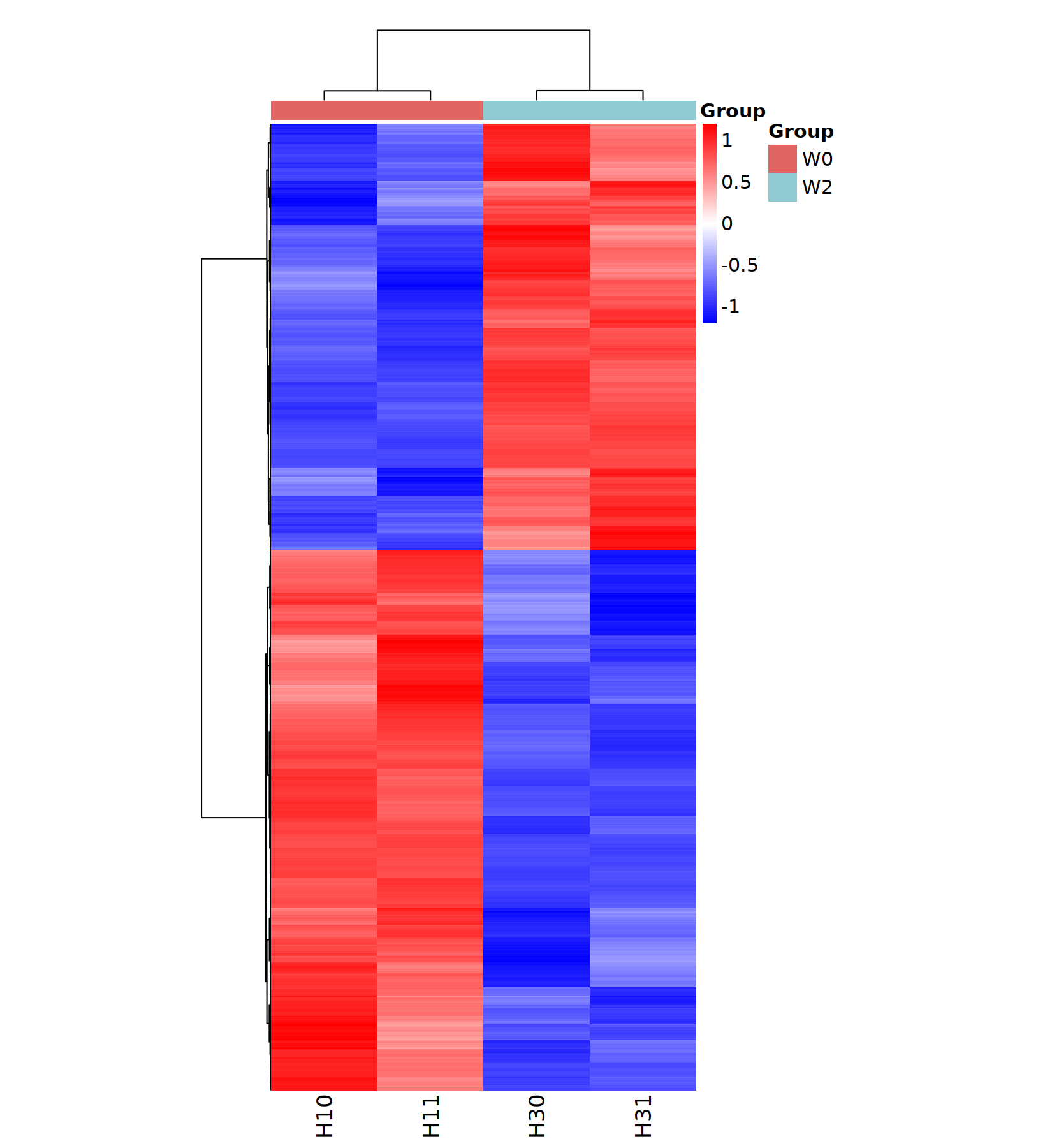


(b)

Supplement: S9 Fig — (DOCX) [file pone.0312416.s009.docx]

**S11 Fig. Statistic of differently expressed metabolite**


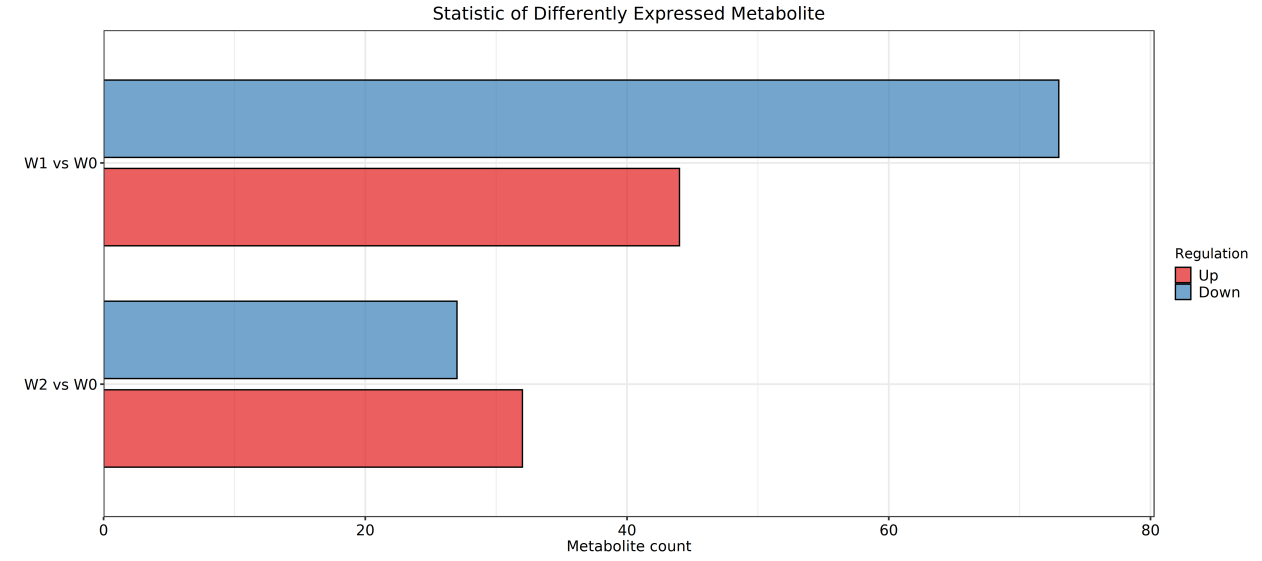

Supplement: S11 Fig — (DOCX) [file pone.0312416.s011.docx]
