# Supplementary material for: Two types of microorganisms isolated from petroleum hydrocarbon pollutants: Degradation characteristics and metabolic pathways analysis of petroleum hydrocarbons
Source: PLoS One. 2024 Nov 13;19(11):e0312416. doi: 10.1371/journal.pone.0312416 (PMC11559972; doi:10.1371/journal.pone.0312416)
Supplement: S10 Fig — (DOCX) [file pone.0312416.s010.docx]

**S10 Fig. volcano plot of gene expression** **(a: strain W01, b: strain W02)**


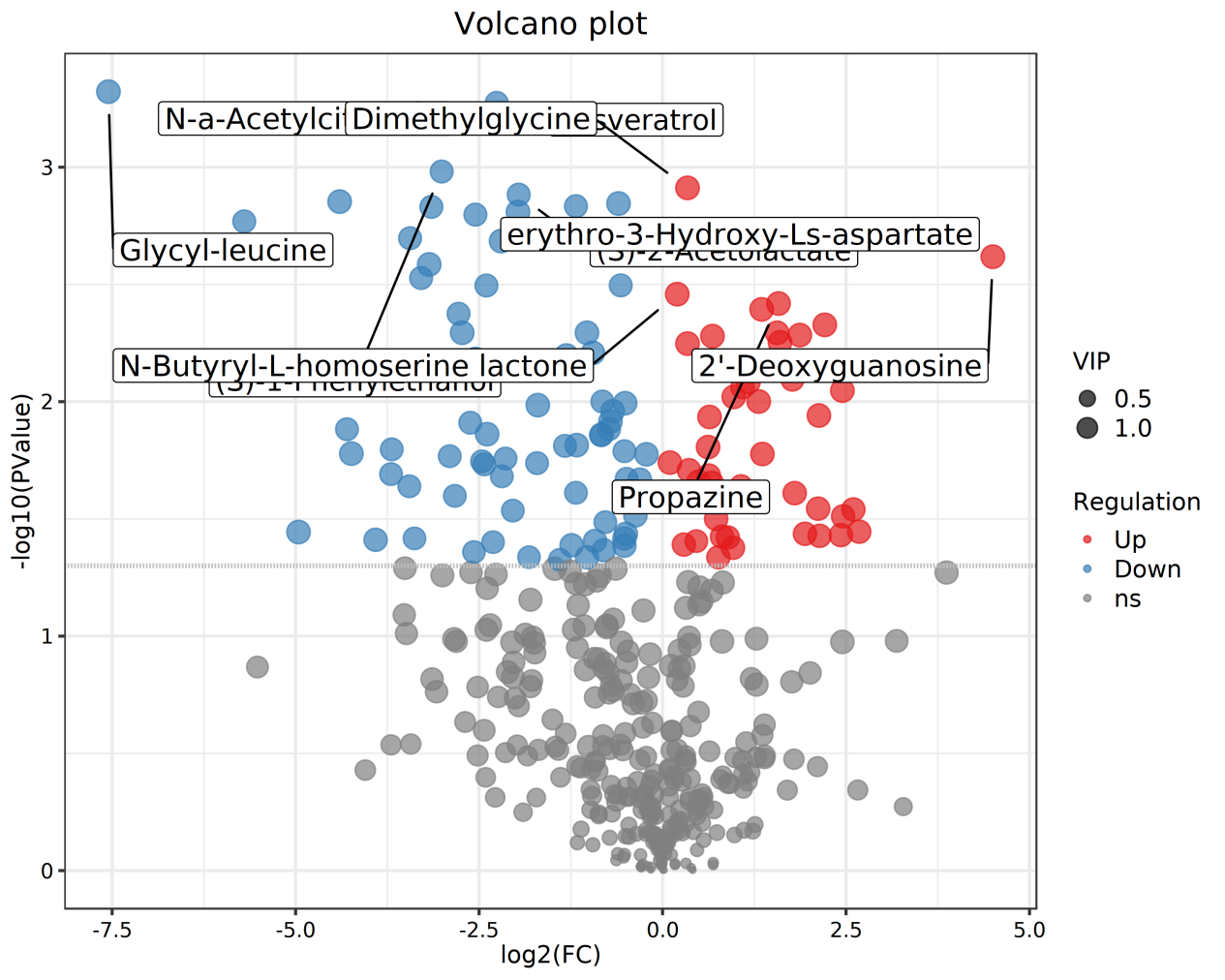


(a)


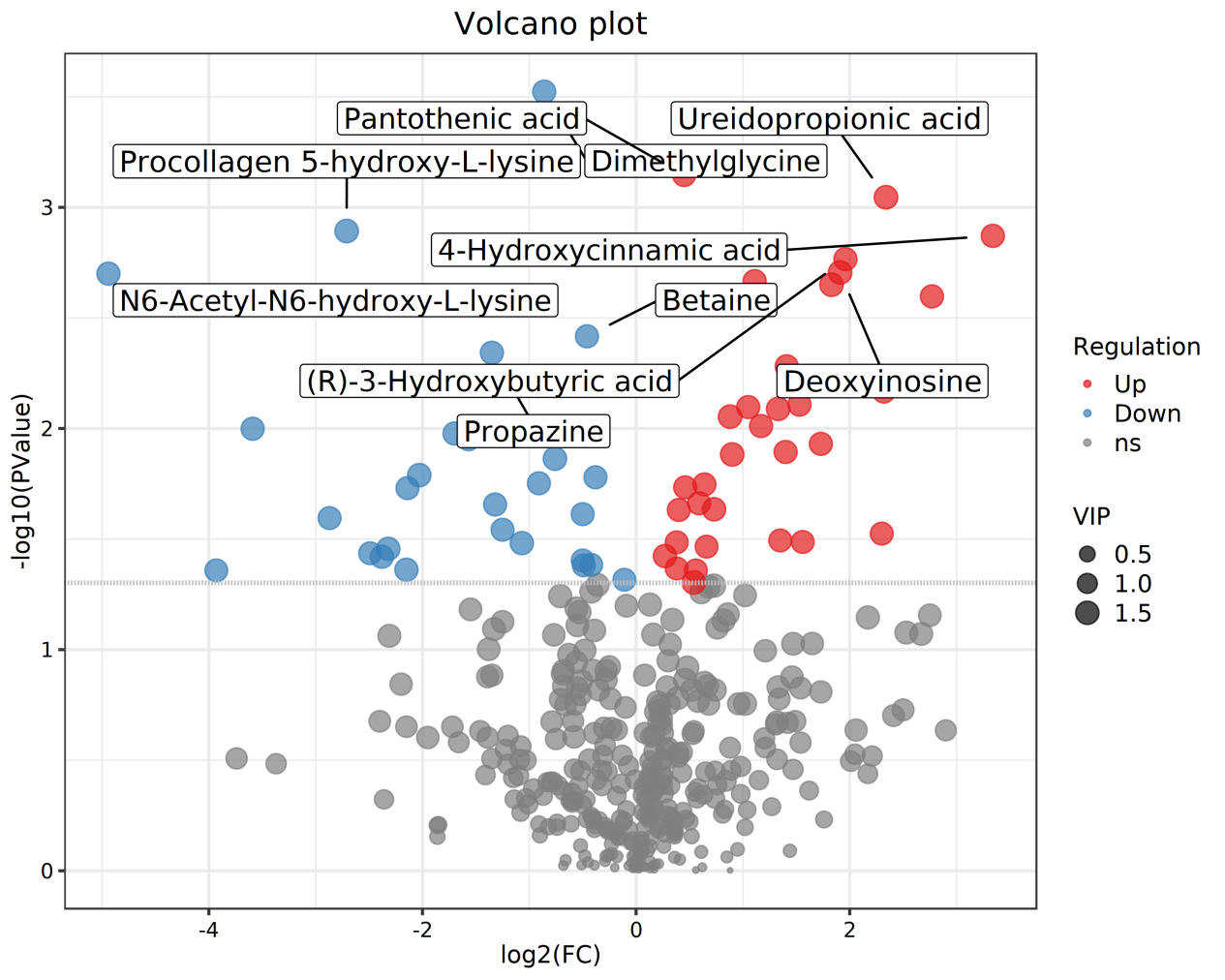


(b)

Note: Genes with significant differential expression are represented by red dots (up regulation) and blue dots (down regulation), while gray dots represent genes with no significant differential expression; The horizontal axis represents the logarithmic value of the differential expression multiple of genes in different samples; The vertical axis represents the statistical significance check value of the difference in gene expression, which is the negative Log10 of FDR (False Discovery Rate).
