# Supplementary material for: Two types of microorganisms isolated from petroleum hydrocarbon pollutants: Degradation characteristics and metabolic pathways analysis of petroleum hydrocarbons
Source: PLoS One. 2024 Nov 13;19(11):e0312416. doi: 10.1371/journal.pone.0312416 (PMC11559972; doi:10.1371/journal.pone.0312416)
Supplement: S1 Section — (DOCX) [file pone.0312416.s012.docx]

**S1 Section Screening, isolation, and purification of bacterial strains**

Strain screening and domestication: Take 1 g of petroleum contaminated soil and 5 mL of activated sludge and add them to 100 mL of domestication medium. Place them in a shaking bed at 30°C and 140 r/min for 5 days, then transfer 4 mL of suspension from them and add them to fresh domestication medium for further cultivation. Repeat domestication 6 times.

Strain separation: Take the bacterial solution from repeated domestication and cultivation for 6 times, dilute it and apply it to a solid LB medium plate. Cultivate it in a constant temperature incubator at 37℃ for 24 hours. Then, select single colonies of different shapes and colors and culture them continuously on a new solid LB medium plate for 3 times. Take the isolated single colonies and place them in domestication medium. After 5 days of cultivation on a shaking bed at 37℃ and 140 r/min. Take 5 mL of culture medium and continue to cultivate in fresh domesticated medium, repeating the separation process 5 times.

Strain purification: Take the above culture medium to prepare diluents of 10^-1^, 10^-2^, 10^-3^, 10^-4^, 10^-5^, and 10^-6^, apply them on LB solid culture medium, invert and culture for 1-4 days, observe the growth of colonies every day, and obtain pure single colonies.
