# Supplementary material for: Two types of microorganisms isolated from petroleum hydrocarbon pollutants: Degradation characteristics and metabolic pathways analysis of petroleum hydrocarbons
Source: PLoS One. 2024 Nov 13;19(11):e0312416. doi: 10.1371/journal.pone.0312416 (PMC11559972; doi:10.1371/journal.pone.0312416)
Supplement: S2 Section — (DOCX) [file pone.0312416.s013.docx]

**S2 Section The testing method of GC-MS**

Analyze using gas chromatography-mass spectrometry (Agilent 7890A-5975C GC-MS).

Pretreatment (He et al., 2008): Take 0.5 mL of oil sample, dilute it 20 times with TCM, and the processed state is uniform and transparent. Then, remove water and pass it through the membrane for machine testing. GC conditions: inlet temperature of 250℃, gas interface temperature of 280℃, carrier gas flow rate of 1.5 mL/min, and split ratio of 4:1.

Heating program: initially 50℃, maintain for 1 minute, and raise the temperature from 5℃/min to 100℃ for 2 minutes; Heat up to 180℃ at 4℃/min and maintain for 3 minutes; Heat up to 250℃ and maintain for 5 minutes at 5℃/min.

MS conditions (Demurtas et al., 2020): ion source temperature 230℃, fourth pole temperature 150℃, EI ionization 70 eV, full scan 35-550 da.

**References**

He Z, Huang Y L, Manohar A K, et al. Effect of electrolyte pH on the rate of the anodic and cathodic reactions in all air-cathode microbial fuelcell[J]. Bioelectrochemistry, 2008, 74(1): 78-82.

Demurtas A, Pescina S, Nicoli S, et al. Validation of aHPLC-UV method for the quantification of budesonide in skin layers[J]. Journal of Chromatography B, 2020, 1164: 122512.
