# Supplementary material for: Two types of microorganisms isolated from petroleum hydrocarbon pollutants: Degradation characteristics and metabolic pathways analysis of petroleum hydrocarbons
Source: PLoS One. 2024 Nov 13;19(11):e0312416. doi: 10.1371/journal.pone.0312416 (PMC11559972; doi:10.1371/journal.pone.0312416)
Supplement: S3 Section — (DOCX) [file pone.0312416.s014.docx]

**S3 Section The toxicity analysis method for intermediate products**

Using the development of toxicity assessment software tool (TEST), the toxicity of compounds was obtained by analyzing known Quantitative Structure Activity Relationship (QSAR) models, specialized literature published by the US EPA and the Inter Agency Testing Committee (ITC), and the results were output.
